# Multiorgan Perfusion by PET: Potential Multiple Pathophysiology Interacting with the Heart to Guide Systemic and Cardiac Management

**DOI:** 10.2967/jnumed.126.272071

**Published:** 2026-07

**Authors:** K. Lance Gould, Nils P. Johnson

**Affiliations:** Weatherhead PET Center, McGovern Medical School, University of Texas, Houston, Texas

In this issue of *The Journal of Nuclear Medicine*, Kärpijoki et al. (1) at Turku PET Center, Finland, under the leadership of Juhani Knuuti extend their long-standing leadership in coronary physiology (2,3) into systemic multiorgan perfusion PET. In the study (1), 191 low-risk patients without brain or coronary artery disease underwent quantitative multiorgan perfusion imaging before and after adenosine stress using long–axis-field-of-view PET and ^15^O-water. With adenosine stress, myocardial, liver, duodenum, and colon perfusion significantly increased while brain, renal, skeletal muscle, bone, and spleen perfusion significantly decreased with no change in pancreas perfusion.

## POTENTIAL VALUE, LIMITED LITERATURE: HOW TO ENVISION WHAT IS NOT COMMON KNOWLEDGE?

The paper demonstrates the feasibility of complex methodology and multiorgan perfusion in a low-risk population (1). Further research needs to address interactive multiorgan perfusion during adenosine versus dobutamine versus supine PET exercise in diverse populations of diffuse and focal systemic and coronary atherosclerosis, myocardial and systemic microvascular disease, diabetes, renal failure, obesity, hypertension, associated nonischemic cardiomyopathy, and heart failure. In these conditions, heart and multiorgan perfusion may provide new insights on coronary and systemic endothelial function, stress hyperemia, microvascular function, and treatment currently undefined.

The Turku PET Center has led clinical application of fundamental concepts in clinical coronary pathophysiology emphasizing that quantitative stress myocardial perfusion predominantly guides invasive interventions after screening CT angiography, not angiographic anatomy (2,3). In the absence of literature or from the low-risk group of the Kärpijoki paper (1), we have only complex multiple pathophysiology of the heart using ^82^Rb to illustrate the potentially important concept using ^15^O-water in a low-risk population. Since editorial words are not adequate for explaining what has not been seen by readers, reviewers, authors, or literature for high-risk populations where the concept is most relevant, we resort to figures of cardiac images to support the concept.

Most inpatients at our tertiary referral and heart failure center have multiorgan pathophysiology impacting the heart and body for which comprehensive coronary flow capacity (CFC) by PET with ^82^Rb quantifies diffuse and focal coronary atherosclerosis, myocardial and systemic microvascular disease of diabetes, renal failure, obesity, hypertension, associated nonischemic cardiomyopathy, with or without focal size severity of artery-specific objective ischemia, and left ventricular function for guiding management and interventions (4,5). For each of the following cardiac PET examples affected by systemic multiple pathophysiology, the readers should ask, what insight would whole-body multiorgan of PET perfusion using ^15^O-water reveal for better understanding or management?

## CASE EXAMPLES MAKE THE CASE

**FIGURE 1.**
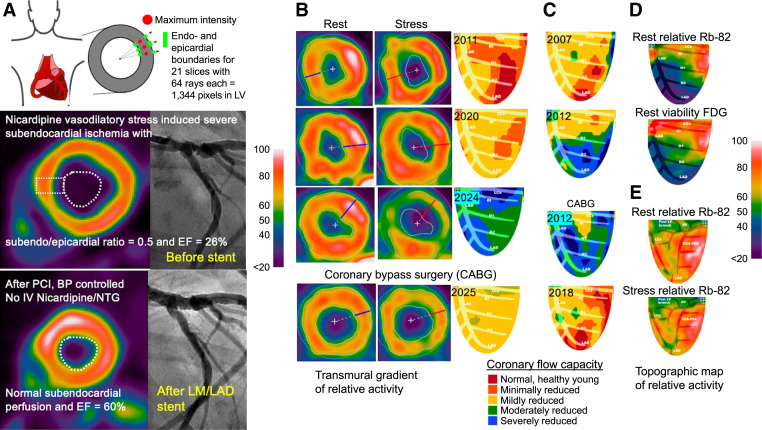
(A) 68-y-old woman in hypertensive crisis, heart failure, atrial fibrillation, EF of 25% by ECHO and chronic kidney disease with blood pressure of 210/80 mm Hg, heart rate of 114/min, cardiac output index of 1.5 L/min/M^2^ while on 5 mg/h of intravenous nicardipine and 140 μg/min of nitroglycerin (NTG) 140 µg/min, mean transmural myocardial perfusion of 1.6 mL/min/g, severe subendocardial ischemia, relative subendo/subepicardial ratio of 0.5, and EF of 26% by PET. After percutaneous coronary intervention (PCI) of left main (LM) and left anterior descending (LAD) arteries, blood pressure was 143/61 mm Hg, heart rate was 82/min, with no intravenous vasodilators, mean transmural resting perfusion of 1.14 mL/min/g, normal subendocardial perfusion, and EF of 60%. (B) 58-y-old man with prior abnormal thallium stress test, high risk factors, no symptoms, had routine rest–stress PET imaging with ^82^Rb showing adequate CFC with mild nonischemic, mildly reduced stress subendocardial relative perfusion without angina or ST changes consistent with diffuse nonobstructive CAD (2011). Routine follow-up PET scans showed initial mild progression (2020), followed by severe progression with angina, severely reduced CFC (2024), >3-mm ST depression during PET stress, and rest–stress EF falling from 63% to 55% during PET stress. Coronary bypass graft surgery (CABG) relieved exertional angina and dyspnea. Routine follow-up PET (2025) showed improvement but diffuse global, mildly reduced CFC (yellow) due to diffuse microvascular dysfunction related to ongoing obesity, past smoking, left ventricle hypertrophy and hypertension. (C) 61-y-old man with prior stent to left anterior descending artery for angina, high-risk factors, and poor medical adherence was referred for serial routine follow-up PET (2007) that showed progression to severe CFC with angina during dipyridamole stress and EF of 40% (2012), leading to CABG with relief of angina. However, postoperative PET showed no improvement in CFC due to residual severe diffuse CAD and severe microvascular disease of diabetes (2012 after coronary artery bypass). After starting on 80 mg of atorvastatin daily with first effective risk factor treatment, PET 6 y later showed dramatic improvement (2018) (all caffeine-negative blood tests). However, EF remained at 39% at rest and 37% at stress, comparable to 40% before CABG, both reduced out of proportion to small subendocardial scar indicating a component of nonischemic cardiomyopathy due to diabetes in addition to CAD with patent bypass grafts and adequate CFC after medical treatment that improved microvascular dysfunction and diffuse CAD. (D) 58-y-old man referred for PET viability after prior percutaneous coronary intervention, myocardial infarction, ventricular tachycardia arrest requiring extracorporeal membrane oxygenation, multiple hospitalizations for heart failure, implanted defibrillator, no angina, and current angiogram showing left circumflex stenosis and in-stent restenosis of right coronary artery. Rest ^82^Rb and FDG imaging showed mixed predominant transmural scar with small border zone of nontransmural scar comprising 68% of the left ventricle, consistent with low EF of 20% by PET blood pool imaging. Low EF is proportionate to large scar in contrast to next example. (E) 76-y-old man referred for rest–stress PET to evaluate cardiac ischemia as cause of low EF. Relative rest–stress perfusion images showed small basal inferolateral nontransmural scar, not worse with stress in distribution of small posterior left ventricle extension branch off right coronary artery comprising ≤10% of the left ventricle. EF of 21% by electrocardiogram-gated PET blood pool imaging (and ECHO) is reduced out of proportion to size of scar or ischemia indicating predominant nonischemic cardiomyopathy of diabetes, hypertension, and renal failure associated with severe microvascular disease and bystander CAD not contributing to low EF.

In Figure 1A, as an example of systemic–coronary physiologic interactions, intravenous nicardipine is a powerful systemic vasodilator for treating hypertensive crisis. However, its parallel powerful coronary vasodilation (6) constitutes a stress test with coincident moderate left main stenosis and diffuse coronary artery disease (CAD), causing severe global subendocardial ischemia and stunned myocardium with low ejection fraction (EF). What would a whole-body scan with ^15^O-water reveal about brain, kidneys, liver, and spleen?

In Figure 1B, due to abnormal thallium stress test with high-risk factors and dense coronary calcium, serial routine PET images showed adequate CFC with mildly reduced subendocardial relative perfusion without angina, indicating mild diffuse nonobstructive CAD. Follow-up PET showed mild progression of diffuse CAD and persisting mildly reduced stress subendocardial perfusion without ischemia. Due to new exertional angina, the PET image from 2024 showed severe CFC progression, ST depression, and falling rest–stress EF. Bypass surgery relieved angina and improved CFC but remained mildly reduced diffusely, indicating diffuse microvascular disease. Would multiorgan PET imaging reveal progressive systemic atherosclerosis or microvascular disease?

Figure 1C illustrates comparable complex disease of progressive CAD and microvascular disease not improved after bypass surgery due to severe microvascular disease. However, in this case, microvascular dysfunction improved over the subsequent 6 y after bypass on new intense risk factor treatment. Again, dynamic cardiac pathophysiology, bypass surgery, and medical treatment imply substantial systemic parallels needing further research.

In Figure 1D, PET viability with rest ^82^Rb perfusion and ^18^F-FDG imaging showed a mixed predominant transmural scar with a small border zone of nontransmural scar comprising 68% of the left ventricle consistent with an EF of 20% by electrocardiogram-gated PET blood pool imaging. The low EF is proportional to the size of the scar, in contrast to the next case characterizing most patients with low EF, CAD without myocardial scarring, and predominant nonischemic cardiomyopathy.

Figure 1E illustrates a patient with intermittent volume overload heart failure without angina but severe microvascular disease associated with diabetes and renal failure. Rest and stress EFs were 34% that were reduced out of proportion to the small nontransmural scar, indicating predominant nonischemic cardiomyopathy with bystander epicardial CAD not contributing to reduced EF, thereby indicating medical management without interventions. For this patient with combined CAD, microvascular dysfunction, and nonischemic cardiomyopathy reducing EF, what would multiorgan PET reveal?

## CONCLUSION

The current study with long–axial-field-of-view PET and ^15^O-water introduces a potentially new era of research extending advanced quantitative PET illustrated for the heart into systemic interacting multiorgan perfusion pathophysiology to guide management.

## DISCLOSURE

No potential conflict of interest relevant to this article was reported.
